# A *Post-hoc* Study of D-Amino Acid Oxidase in Blood as an Indicator of Post-stroke Dementia

**DOI:** 10.3389/fneur.2019.00402

**Published:** 2019-04-26

**Authors:** Yi-Chun Chen, Wen-Hai Chou, Hsiao-Hui Tsou, Chiu-Ping Fang, Tung-Hsia Liu, Hsien-Hao Tsao, Wen-Chuin Hsu, Yi-Chinn Weng, Yun Wang, Yu-Li Liu

**Affiliations:** ^1^Department of Neurology, Chang Gung Memorial Hospital Linkou Medical Center and College of Medicine, Chang Gung University, Taoyuan, Taiwan; ^2^Dementia Center, Chang Gung Memorial Hospital, Taoyuan, Taiwan; ^3^Center for Neuropsychiatric Research, National Health Research Institutes, Miaoli, Taiwan; ^4^Division of Biostatistics and Bioinformatics, Institute of Population Health Sciences, National Health Research Institutes, Miaoli, Taiwan; ^5^Graduate Institute of Biostatistics, China Medical University, Taichung, Taiwan; ^6^Department of Medicine, Chang Gung University, Taoyuan, Taiwan

**Keywords:** D-amino acid oxidase (DAO), stroke, dementia, biomarker, post-stroke dementia, cerebral infarction

## Abstract

Stroke is an important risk factor for dementia. Epidemiological studies have indicated a high incidence of dementia in stroke patients. There is currently no effective biomarker for the diagnosis of post-stroke dementia (PSD). D-amino acid oxidase (DAO) is a flavin-dependent enzyme widely distributed in the central nervous system. DAO oxidizes D-amino acids, a process which generates neurotoxic hydrogen peroxide and leads to neurodegeneration. This study aimed to examine post-stroke plasma DAO levels as a biomarker for PSD. In total, 53 patients with PSD, 20 post-stroke patients without dementia (PSNoD), and 71 age- and gender-matched normal controls were recruited. Cognitive function was evaluated at more than 30 days post-stroke. Plasma DAO was measured using the enzyme-linked immunosorbent assay. White matter hyperintensity (WMH), a neuroimaging biomarker of cerebral small vessel diseases, was determined by magnetic resonance imaging. We found that plasma DAO levels were independently higher in PSD subjects than in PSNoD subjects or the controls and were correlated with the WMH load in stroke patients. Using an area under the curve (AUC)/receiver operating characteristic analysis, plasma DAO levels were significantly reliable for the diagnosis of PSD. The sensitivity and specificity of the optimal cut-off value of 321 ng/ml of plasma DAO for the diagnosis of PSD were 75 and 88.7%, respectively. In conclusion, our data support that plasma DAO levels were increased in PSD patients and correlated with brain WMH, independent of age, gender, hypertension, and renal function. Plasma DAO levels may therefore aid in PSD diagnosis.

## Introduction

Stroke is a risk factor for both vascular dementia and Alzheimer's disease (1, 2). Functional recovery develops over the course of 26 weeks after a stroke (3), but the survivors are often left with disabilities. In addition to the sequelae of acute neuronal damage, the 1-year post-stroke dementia (PSD) rates after first-ever and recurrent stroke are ~10 and 30%, respectively (4). Although there is a high risk of PSD in chronic stroke patients, no effective biomarker has been established for PSD (5). Limited studies have indicated that PSD involves secondary degeneration (6), including neuronal death, axonal degeneration, inflammation, and gliosis (7, 8). These secondary neurodegenerative changes result in Wallerian degeneration (9), cortical atrophy (9), white matter hyperintensities (WMHs) (9–11), or lacunes (9–11), and have been used as neuroimaging biomarkers for vascular brain injury (12) and cognitive impairment (13). The value of these indicators in predicting PSD is, however, still uncertain (11). A few blood biomarkers [e.g., β-secretase (14), homocysteine (15), and inflammation markers (16–18)] for predicting PSD have been examined. However, the use of these biomarkers for clinical PSD patients is limited by their specificity and sensitivity. Currently, there are no effective blood biomarkers for PSD (2).

D-amino acid oxidase (DAO), a peroxisome flavin-dependent oxidase, catalyzes the stereospecific oxidative deamination of D-serine to imino-serine, hydrogen peroxide, and ammonia, which lead to oxidative stress (19). DAO expression and D-serine catabolism is widespread in different brain regions (20). Areas enriched in D-serine include the cerebral cortex, hippocampus, thalamus, hypothalamus, and amygdala (21). Since ischemic injury induces peroxisome biogenesis in neurons (22), DAO expression may also be regulated after stroke. This study aimed to examine post-stroke plasma DAO levels and determine whether they can be used as a biomarker for PSD.

## Materials and Methods

### Subjects

This study was carried out in accordance with the recommendations of Chang Gung Memorial Hospital Institution Ethics Review Board with written informed consent from all subjects. All subjects gave written informed consent in accordance with the Declaration of Helsinki. The protocol was approved by the Chang Gung Memorial Hospital Institution Ethics Review Board (201600197B0) and the Research Ethics Committee of the National Health Research Institutes (EC1051105-E). Patients with ischemic stroke were enrolled at the Chang Gung Memorial Hospital.

Ischemic stroke and its subtypes were diagnosed and classified according to their clinical presentations and patient brain imaging. This study enrolled chronic stroke patients (more than 30 days after the onset of stroke). To decrease the heterogeneity within the ischemic stroke groups, we only included stroke patients with lacunar or atherothrombotic infarction. The diagnosis was based on the Trial of ORG 10172 in acute stroke treatment (TOAST) criteria with modifications (23) as we previously reported (24): (1) lacunar infarction was diagnosed when the brainstem or subcortical hemispheric ischemia was <1.5 cm in diameter on brain imaging without cortical or cerebellar dysfunction, and (2) atherothrombotic infarction was diagnosed when the ischemic lesion was >1.5 cm and arterial stenosis was >50% of any of the carotid/cerebral artery. Stroke patients with cardioembolic infarction, atrial fibrillation, acute coronary syndrome, end-stage renal disease, infection, or inflammatory disease, prior neurodegenerative disease, undetermined stroke type, or cognitive deficit prior to stroke were excluded (25). Healthy control subjects with no history of stroke, neurodegenerative disease, overt medical disease, or cancer were recruited randomly from the community.

Stroke patients who had no cognitive complaints were classified into the group of post-stroke without dementia (PSNoD) after evaluation by two neurologists. PSD was diagnosed by two neurologists according to the requirements of the National Institute of Neurological Disorders and Stroke-Association Internationale pour la Recherche et l'Enseignement en Neurosciences (NINDS-AIREN) criteria for probable vascular dementia (25), along with the temporal relationship between the stroke event and cognitive decline, confirmation of atherothrombotic, or lacunar infarction on brain imaging, evidence of prominent executive dysfunction, a clinical dementia rating equal to or greater than 0.5 (to influence activities of daily living), and a Mini-Mental State Examination (MMSE) score lower than 22 for literate individuals and 20 for illiterate individuals (26).

### Clinical Information

Anthropometric data and 12-h fasting blood samples were collected from all participants. Hypertension (HTN) was defined as the use of antihypertensive drugs, an average of three independent measures of systolic blood pressure of 140 mmHg or greater, or of diastolic blood pressure of 90 mmHg or greater. Diabetes mellitus (DM) was defined based on the World Health Organization criteria (27).

### WMH Measurement

Brain magnetic resonance imaging (MRI) was conducted within 1 week post-stroke. Visual rating of WMH severity was determined based on the Scheltens scale on T2-weighted and fluid-attenuated inversion recovery scans (28). The Scheltens scale was summed using 0- to 6-point severity ratings in the periventricular (frontal caps, bands, and occipital bands), deep (frontal, temporal, parietal, occipital), basal ganglia (caudate, putamen, globus pallidus, thalamus, internal/external capsule), and infratentorial areas (cerebellum, mesencephalon, pons, medulla) (29).

### Plasma DAO Assay

Plasma DAO was measured using an enzyme-linked immunosorbent assay (ELISA) kit (Biomatik, USA). This assay employs the quantitative double-antibody sandwich enzyme immunoassay technique. Antibody specific for DAO was pre-coated onto a microplate. Plasma was diluted 10 times with Dulbecco's phosphate-buffered saline. A 100-μl volume of standard or sample was pipetted into each well and incubated for 2 h at 37°C. The standard or sample was aspirated and 100 μl of detection reagent A, biotin-conjugated antibody specific for DAO, was added to each well and incubated at 37°C for 1 h. The samples were washed three times with 1X wash solution to remove any unbound reagent, and then 100 μl of detection reagent B, avidin-conjugated horseradish peroxidase (HRP), was added and incubated for 30 min at 37°C. After another 5 washings, 90 μl of substrate solution was added, and incubated at 37°C. During this reaction, color develops in proportion to the amount of DAO bound on the plate. Color development was stopped at 20 min by adding 50 μl of stop solution. The intensity of the color was measured immediately by SpectraMax M2e multi-mode microplate reader (Molecular Devices, CA, USA) at a wavelength of 450 nm. After subtracting the values for blank, the semi-log curve fitting and analysis of data was performed using GraphPad Prism 5 (GraphPad Software, Inc., CA, USA).

### Statistics and Power Estimation

Comparisons between patients with ischemic stroke and normal controls were conducted by the Kruskal-Wallis test, Mann-Whitney *U*-test, or chi-square test, and Fisher's exact test, where appropriate. General linear model or logistic regression analyses were used to evaluate covariates associated with age and gender. *Post hoc* comparisons employed least square means. Spearman's correlation analysis was used to test the associations between plasma DAO and age, eGFR, WMH or MMSE. Normal distributions for continuous data were tested by the Shapiro-Wilk test. The independent variables were tested for their abilities to predict the plasma DAO levels by multiple regression analysis. The receiver operating characteristic (ROC) curve was used to calculate the sensitivity and specificity of plasma DAO in predicting PSD. The significance level was set at *P* < 0.05. A power >0.8 was used to detect a difference in DAO levels between the PSD and PSNoD groups. All statistical analyses were performed using SAS software, Version 9.4 (SAS Institute, Inc., Cary, NC) and plotted by GraphPad Prism 5 (GraphPad Software, Inc., CA, USA).

## Results

### Characteristics of Patients With Ischemic Stroke

In total, 20 PSD, 53 PSNoD patients, and 71 age- and gender-matched normal controls (NC) were recruited (Table 1 and Supplementary Table 1). Their average age was 64 years with 71% being male. The PSD patients were older than the controls and the PSNoD patients. High incidences of HTN and DM and low estimated glomerular filtration rates (eGFRs) were found in the PSNoD and PSD groups. In the stroke patients, the average post-stroke time was 3.14 ± 3.18 years with a significant difference between PSNoD and PSD groups when adjusted for age and gender (*P* = 0.046). The average functional outcome at 30 days after stroke, rated by the Modified Rankin scale (MRS), was 0.92 ± 0.92 in stroke patients; no significant difference was found between PSNoD (0.79 ± 0.77), and PSD (1.50 ± 1.31) patients after adjusting age and gender (*P* = 0.08). The WMHs on MRI were similar in those with and without post-stroke dementia. The PSD group had a significantly lower MMSE score (16.15 ± 6.64, *P* < 0.0001) when compared to NC (27 ± 2), or PSNoD patients (26.13 ± 2.83). There was no difference in the average total WMH score between PSNoD and PSD patients (8.46 ± 4.47 and 11.92 ± 6.29, respectively).

**Table 1:** Association analyses among ischemic stroke patients with or without dementia and normal controls.

**Variable**	**Normal Control**	**PSNoD**	**PSD**	**Unadjusted/Adjusted *P*-value**
	***N*, Mean ± SD**	***N*, Mean ± SD**	***N*, Mean ± SD**	
Age	71, 64.03 ± 10.81	53, 61.7 ± 8.95	20, 69.35 ± 7.24	**0.014 ^†^, ^‡^**
Gender				0.17
Female	21 (29.58%)	12 (22.64%)	9 (45.00%)	
Male	50 (70.42%)	41 (77.36%)	11 (55.00%)	
HTN				**0.003^†^/0.010^†^**
No	30 (43.48%)	14 (26.42%)	1 (5.00%)	
Yes	39 (56.52%)	39 (73.58%)	19 (95.00%)	
DM				0.001 ^*^, ^†^/0.002 ^*^, ^†^
No	52 (75.36%)	27 (50.94%)	7 (35.00%)	
Yes	17 (24.64%)	26 (49.06%)	13 (65.00%)	
eGFR (ml/min/1.73 m^2^)	64, 94.27 ± 25.17	53, 83.9 ± 24.91	20, 74.55 ± 29.13	0.011^*^, ^†^/0.009^*^, ^†^
LDL (mg/dl)	60, 125.32 ± 51.86	53, 128.92 ± 37.01	18, 135.89 ± 44.61	0.42/0.77
MRS	0, –	53, 0.79 ± 0.77	12, 1.50 ± 1.31	0.07^§^/0.08
Post-stroke time (years)	0, –	53, 2.63 ± 2.83	20, 4.47 ± 3.72	**0.028^§^/0.046**
MMSE score	17, 27 ± 2	16, 26.13 ± 2.83	20, 16.15 ± 6.64	**<0.0001^†^,^‡^/ < 0.0001^†^,^‡^**
Education (years)	38, 7.45 ± 4.06	45, 8.73 ± 4.15	20, 4.75 ± 4.42	**0.005^†^,^‡^/0.08**
WMH-MRI (Scheltens scale)	0, –	26, 8.46 ± 4.47	13, 11.92 ± 6.29	0.052^§^/0.12
DAO (ng/ml)	71, 217.21 ± 83.6	53, 265.04 ± 106.42	20, 359.16 ± 95.76	<0.0001 ^*, †, ‡^/ < 0.0001^*, †, ‡^
Stroke subtypes				0.49/0.88
L	0, –	26 (49.06%)	8 (40.00%)	
A	0, –	27 (50.94%)	12 (60.00%)	
Stroke loci				0.47/0.07
D	0, –	42 (79.25%)	16 (80.00%)	
C	0, –	9 (16.98%)	2 (10.00%)	
D+C	0, –	2 (3.77%)	2 (10.00%)	

DAO, D-amino acid oxidase; DM, diabetes mellitus; HTN, hypertension; LDL, low-density lipoprotein; MMSE, mini-mental state examination; MRS, Modified Rankin scale; PSD, Post-stroke dementia; PSNoD, Post-stroke without dementia; SD, Standard Deviation; WMH-MRI, White matter hyperintensity on magnetic resonance imaging. Adjusted P-value: General linear model or logistic regression analysis, adjusted for age, and gender. Stroke subtypes: L, lacunar infarction or A, atherothrombotic infarction. Stroke loci: D, deep; C, cortical; D+C, deep + cortical. Kruskal-Wallis test in 3 groups for continuous data and chi-square test or Fisher's exact test for categorical data. Bold values indicate P < 0.05.

§ *Mann-Whitney U-test in 2 groups for continuous data*.

*, †, and ‡ *The symbol for multiple comparisons (P < 0.05)*.

* *Normal Control vs. PSNoD*.

† *Normal Control vs. PSD*.

‡ *PSNoD vs. PSD*.

### Increased Plasma DAO Levels in Patients With PSNoD or PSD

**Figure 1:**
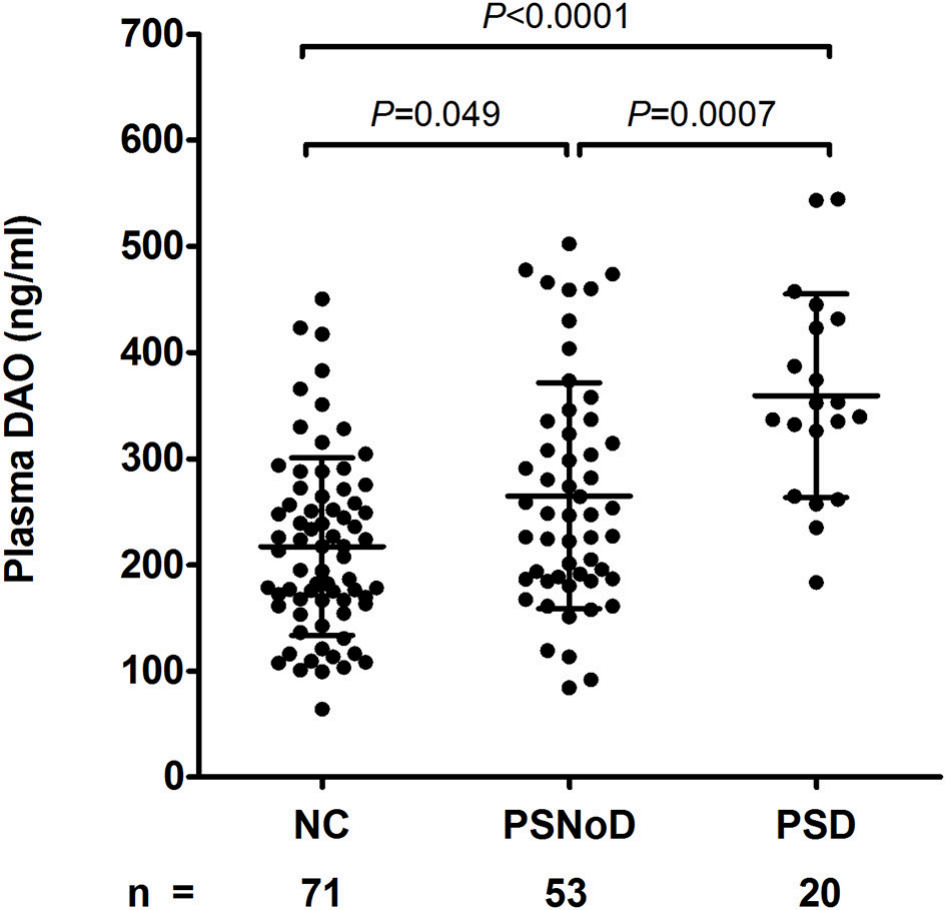
Plasma D-amino acid oxidase levels (DAO, ng/ml) were different between post-stroke without dementia (PSNoD) and post-stroke dementia (PSD) patients, and normal controls (NC), adjusted for age, gender, and eGFR.

Plasma DAO levels were higher in the stroke patients (291 ± 111 ng/ml) than in the NC (217 ± 84 ng/ml) (Mann-Whitney *U*-test, *P* < 0.0001) (Supplementary Table 1). The highest DAO level was found in PSD patients (359 ± 96 ng/ml), followed by PSNoD patients (265 ± 106 ng/ml), and NC (217 ± 84 ng/ml, Table 1). The rank order of the plasma DAO level was PSD > PSNoD > NC after adjusting for age, gender, and eGFR (Figure 1), or for age and gender (Supplementary Figure 1).

### Correlation of Plasma DAO With Age, HTN, eGFR, WMH, and MMSE

**Figure 2:**
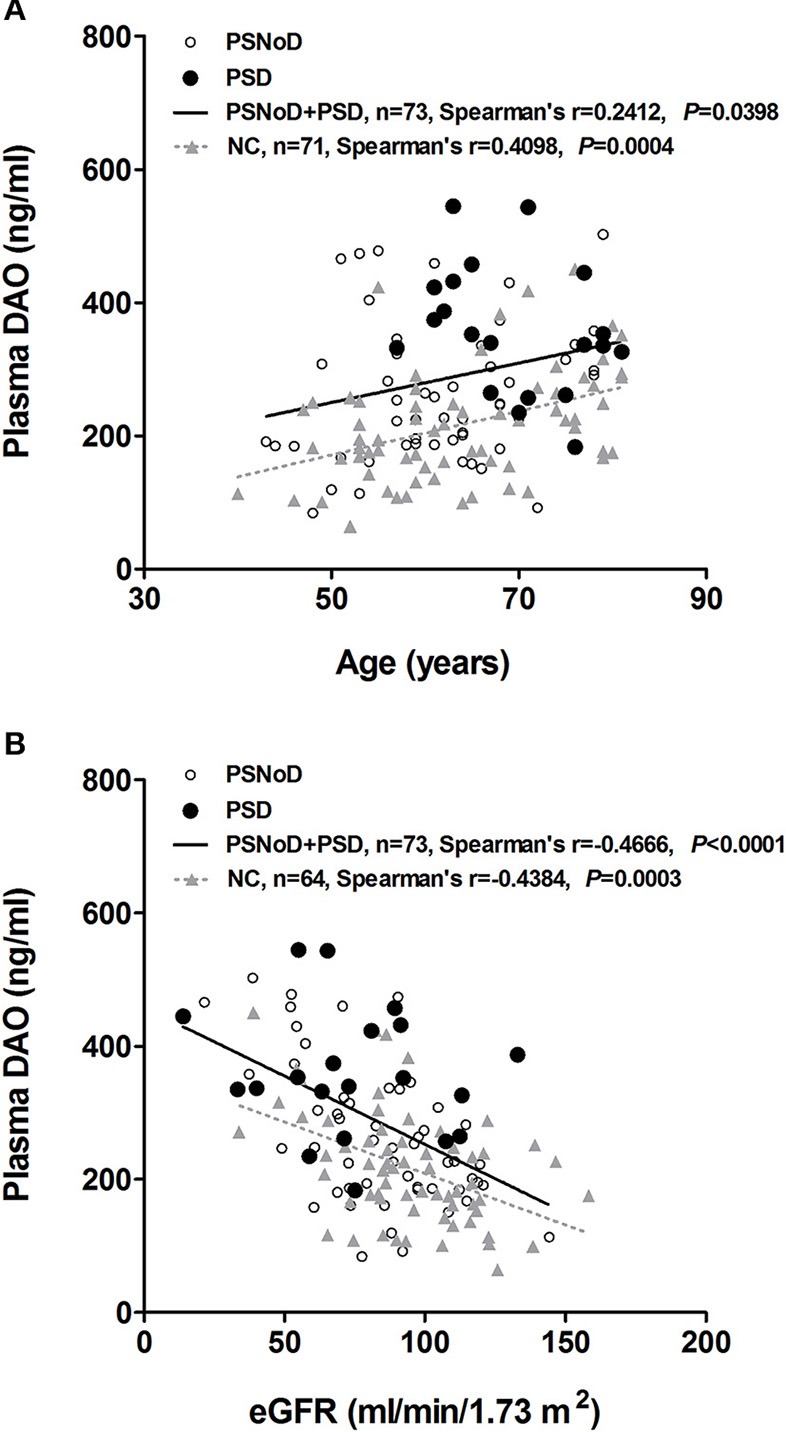
Spearman's correlation and linear trend line **(A)** between plasma D-amino acid oxidase (DAO, ng/ml) and age, and **(B)** between plasma DAO (ng/ml) and eGFR (ml/min/1.73 m^2^) in chronic ischemic stroke and normal control (NC) patients. PSD, Post-stroke dementia; PSNoD, Post-stroke without dementia.

**Figure 3:**
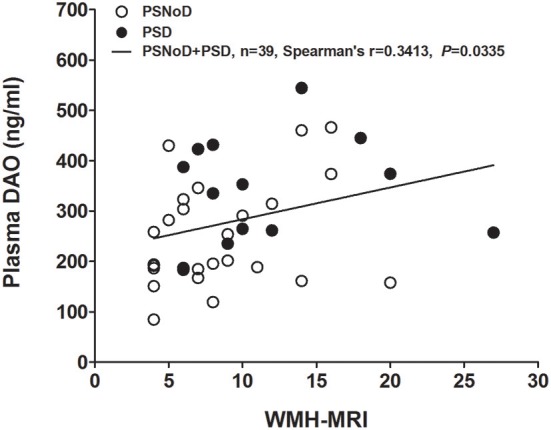
Plasma D-amino acid oxidase levels (DAO, ng/ml) were significantly and positively correlated with the white matter hyperintensity (WMH, total Scheltens scale score) in chronic ischemic stroke patients. PSD, Post-stroke dementia; PSNoD, Post-stroke without dementia.

**Figure 4:**
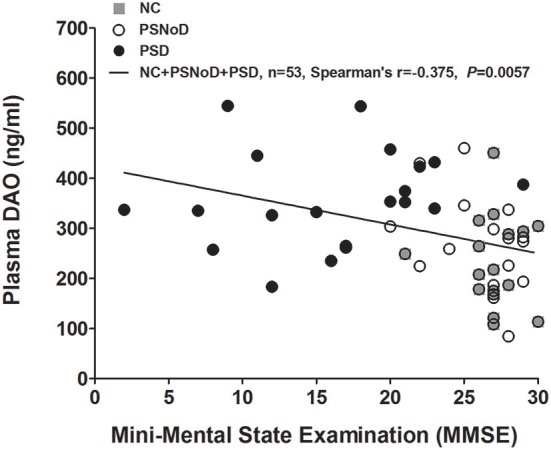
Plasma D-amino acid oxidase (DAO) levels were negatively correlated with Mini-Mental State Examination (MMSE) scores. NC, Normal controls; PSD, Post-stroke dementia; PSNoD, Post-stroke without dementia.

Univariate regression analysis revealed that plasma DAO levels positively correlated with age (β = 3.1, *P* = 0.0003) and with HTN (β = 67.49, *P* = 0.0003), and negatively with eGFR (β = −2.06, *P* = 1.47 X 10^−10^) (Supplementary Table 2). The correlation between DAO and age was prominent in the NC (*r* = 0.41, *P* = 0.0004), as compared to PSNoD and PSD patients (*r* = 0.24, *P* = 0.04) (Figure 2A). Plasma DAO levels negatively correlated with eGFR in stroke patients (*r* = –0.47, *P* < 0.0001) and controls (*r* = –0.44, *P* = 0.0003) (Figure 2B). A trend toward significance was found between DAO and WMH load (*P* = 0.06) in stroke patients. High plasma DAO levels were found in the stroke patients with high WMH loads (*r* = 0.34, *P* = 0.0335) (Figure 3). Plasma DAO negatively correlated with the MMSE score (*r* = –0.375, *P* = 0.0057) (Figure 4).

### Confounding Factor Analysis

Confounding factors were analyzed by multivariate regression analysis. As seen in Table 2, the difference in plasma DAO levels between PSNoD and PSD groups was not affected by age, gender, time post-stroke, renal function, or HTN, suggesting that DAO is an independent marker for PSD.

**Table 2:** Multiple regression of D-amino acid oxidase (DAO, ng/ml) between post-stroke dementia (PSD), and post-stroke without dementia (PSNoD) patients.

**Variable**	**β**	**S.E**.	***t***	***P*-value**	**Partial *r^**2**^***	**VIF**
PSD vs. PSNoD	86.19	26.60	3.24	**0.002**	0.144	1.29
Post-stroke time (years)	2.54	3.54	0.72	0.476	0.013	1.15
Age	−0.28	1.37	−0.20	0.839	0.012	1.43
Gender (Male)	66.31	24.22	2.74	**0.008**	0.079	1.10
eGFR (ml/min/1.73 m^2^)	−1.88	0.44	−4.23	**7.27** **×** **10**^**−5**^	0.161	1.23
HTN (+)	12.75	27.69	0.46	0.647	0.002	1.15

*n = 73, F = 7.68, P < 0.0001, adjusted r^2^ = 35.78%. β, regression coefficient; S.E, standard error of regression coefficient; t, the test statistics on slope of the regression line; VIF, variance inflation factor. Bold values indicate P < 0.05*.

### Optimal Cut-Off Point Calculated by ROC Analysis for PSD Diagnosis

**Figure 5:**
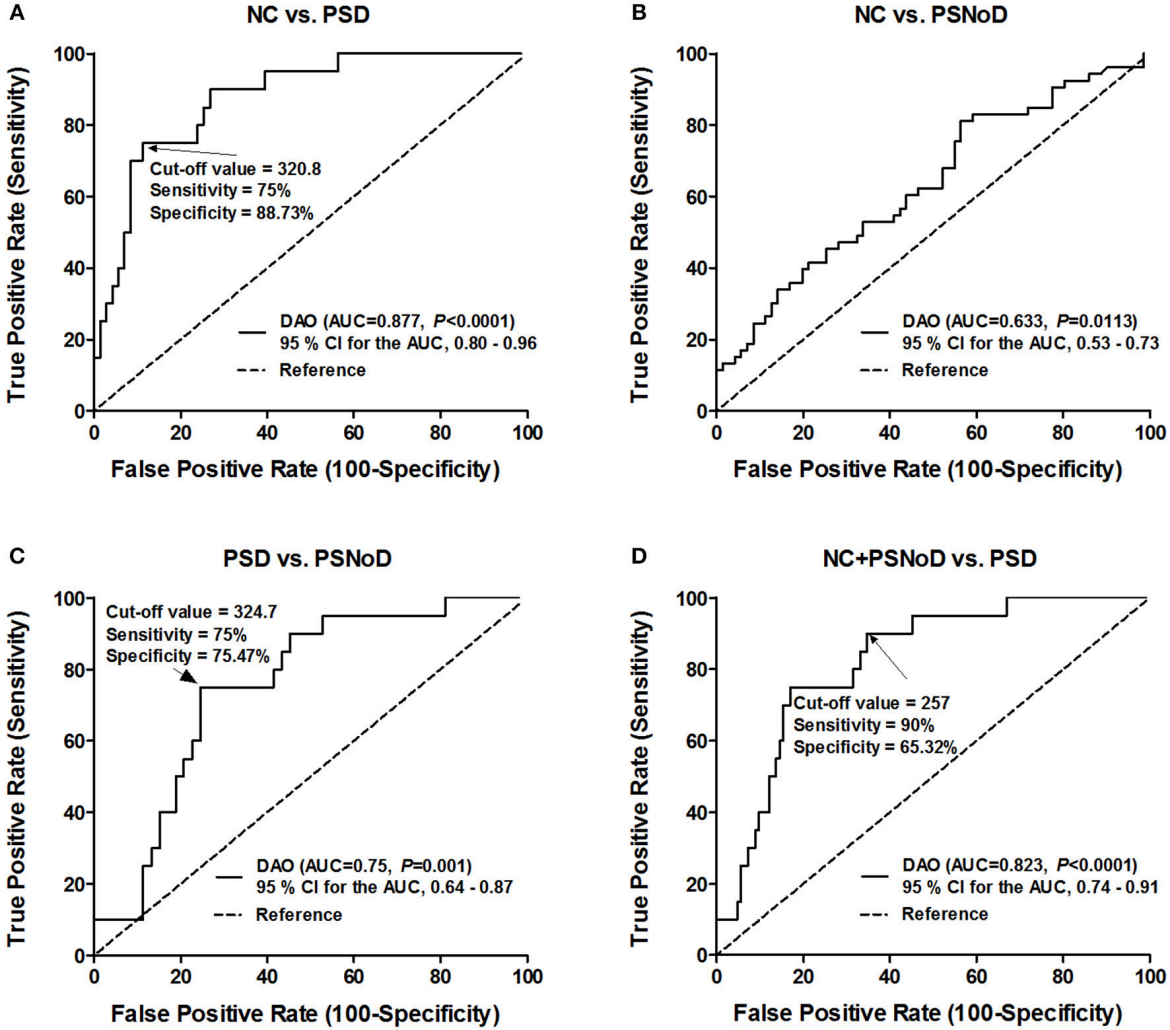
Specificity and sensitivity of using plasma D-amino oxidase (DAO) to diagnose post-stroke dementia (PSD). **(A)** Distinguishing normal controls (NC) from post-stroke dementia patients (PSD). **(B)** There is no cut-off value to distinguish between NC and post-stroke patients without dementia (PSNoD). **(C)** Distinguishing PSD from PSNoD patients. **(D)** Distinguishing NC + PSNoD patients from PSD patients. AUC, Area under the curve; CI, Confidence interval.

In the area under the curve (AUC)/ROC analysis, the level of plasma DAO was significantly correlated with PSD when compared to controls (AUC = 0.877; 95% confidence interval (CI), 0.80–0.96; *P* < 0.0001), PSNoD (AUC = 0.75; 95% CI, 0.64–0.87; *P* = 0.001), or controls + PSNoD (AUC = 0.823; 95% CI, 0.74–0.91; *P* < 0.0001) (Figure 5). The optimal cut-off value of plasma DAO for the diagnosis of PSD is 321 ng/ml (sensitivity = 75%, specificity = 88.7%).

## Discussion

Mounting evidence has demonstrated a high incidence of cognitive impairment, between 6 and 32%, in stroke patients (1, 2). The wide range of reported incidence is partly due to the lack of consistent standards for the diagnosis of PSD. In the present study, the NINDS-AIREN criteria were used to separate PSD from Alzheimer's disease (30). Patients with PSD had the lowest MMSE when compared to PSNoD patients or NC. In contrast, the average MMSE score was similar between controls and PSNoD patients. Plasma DAO levels were significantly elevated in the PSD patients after adjusting for the effects of age, gender, and renal function. Plasma DAO level was moderately correlated with the brain WMH load in the chronic stroke patients, and an elevation of plasma DAO levels was associated with PSD. Our data suggest that DAO is involved in the pathogenesis of chronic stroke and PSD, and that plasma DAO levels can be a biomarker for PSD.

DAO has dual roles in post-stroke change. First, DAO catalyzes D-serine to toxic metabolites (i.e., imino-serine, hydrogen peroxide, and ammonia), leading to oxidative stress (19, 22) and interference with stroke recovery. On the other hand, D-serine metabolites modulate the N-methyl-D-aspartate (NMDA) receptor response in synapses for neuronal survival and synaptic plasticity (4). In this study, we demonstrated that elevation of peripheral DAO levels is associated with the dementia status in chronic stroke patients. Our findings are supported by the increase of peripheral DAO levels in cognitive decline (31, 32). Since DAO is expressed mainly in the peroxisomes (22), high peripheral DAO levels in chronic stroke patients may suggest that peroxisome biogenesis is activated in the chronic stage of stroke.

DAO is concentrated in the kidneys and brain (33). The kidney-brain inflammatory cross-talk in neuropsychiatric disorders, cognitive impairment, and dementia has been noted in epidemiologic studies (34). In this study, we demonstrated a negative correlation between plasma DAO levels and eGFR, which was further supported by the high DAO levels found in patients with chronic renal disease (35, 36). However, after adjusting for eGFR, we found elevated DAO levels in PSD patients, suggesting that the increase in DAO is independent of eGFR and that non-kidney tissues may be involved in the increases in peripheral DAO in PSD patients. To our knowledge, elevated DAO in PSD patients has not previously been reported. Further studies with more patients in different ethnic groups are needed to confirm peripheral plasma DAO as a biomarker for PSD.

This study has several limitations. It is a cross-sectional study, and therefore the exact timeline of cognitive decline is not available. In addition, we did not consider cognitive impairment without dementia, the severity of any depression, or the concurrent medication with NMDA antagonists; these factors may limit our power to illuminate the role of DAO in the deteriorating cognitive process. PSD is diagnosed by clinical presentation, clinical dementia rating, and MMSE, and may be potentially underdiagnosed in patients with very mild executive dysfunction or over-diagnosed in those with depression. A battery of detailed neuropsychological assessments may help define very early vascular cognitive impairment before the occurrence of dementia. Furthermore, WMHs were measured by a visual quality rating scale instead of volumetric quantification. Prospective longitudinal studies with comprehensive neuropsychological tests are needed to strengthen these findings.

In summary, we demonstrated elevated plasma DAO levels in patients with cognitive impairment after stroke. We propose the use of plasma DAO level as a diagnosis biomarker for PSD, along with neuroimaging and clinical presentation.

## Ethics Statement

This study was carried out in accordance with the recommendations of Chang Gung Memorial Hospital Institution Ethics Review Board with written informed consent from all subjects. All subjects gave written informed consent in accordance with the Declaration of Helsinki. The protocol was approved by the Chang Gung Memorial Hospital Institution Ethics Review Board (201600197B0), and the Research Ethics Committee, National Health Research Institutes (EC1051105-E).

## Author Contributions

Y-CC and Y-LL designed the study, obtained funding, and supervised data collection. W-HC, C-PF, H-HTsao, W-CH, and Y-CW contributed to the data collection. H-HTsou, T-HL performed the data analysis. YW revised the final manuscript. All authors read and approved the final manuscript.

### Conflict of Interest Statement

The authors declare that the research was conducted in the absence of any commercial or financial relationships that could be construed as a potential conflict of interest.

## Abbreviations

CI: Confidence interval
DAO: D-amino acid oxidase
PSD: Post stroke dementia
PSNoD: Post-stroke without dementia
NC: Normal control
MMSE: Mini-Mental State Examination
WMH: White matter hyperintensity
MRI: Magnetic resonance imaging
AUC: Area under the curve
AD: Alzheimer's disease
TOAST: Trial of ORG 10172 in acute stroke treatment
LDL: low-density lipoprotein
NINDS-AIREN: Neurological Disorders and Stroke-Association Internationale pour la Recherche et l'Enseignement en Neurosciences
NMDA: N-methyl-D-aspartate
ROC: Receiver operating characteristic
HTN: Hypertension
DM: Diabetes mellitus
eGFR: Estimated glomerular filtration rate
MRS: Modified Rankin scale
SD: standard deviation
SE: standard error
VIF: variance inflation factor.
