# Supplementary material for: A Post-hoc Study of D-Amino Acid Oxidase in Blood as an Indicator of Post-stroke Dementia
Source: Front Neurol. 2019 Apr 26;10:402. doi: 10.3389/fneur.2019.00402 (PMC6497996; doi:10.3389/fneur.2019.00402)
Supplement: Supplementary file 1 [file Data_Sheet_1.doc]

**Supplementary material**

**Supplementary Table 1.** Characteristics between normal controls and ischemic stroke patients.

| Variable | Normal Control |  | Ischemic stroke |  | *P*-value |
| --- | --- | --- | --- | --- | --- |
|  | N, Mean ± SD |  | N, Mean ± SD |  |  |
| Age | 71, 64.03 ± 10.81 |  | 73, 63.79 ± 9.14 |  | 0.998 |
| Gender |  |  |  |  | 0.91 |
| Female | 21 ( 29.58 % ) |  | 21 ( 28.77 % ) |  |  |
| Male | 50 ( 70.42 % ) |  | 52 ( 71.23 % ) |  |  |
| HTN |  |  |  |  | **0.003** |
| No | 30 ( 43.48 % ) |  | 15 ( 20.55 % ) |  |  |
| Yes | 39 ( 56.52 % ) |  | 58 ( 79.45 % ) |  |  |
| DM |  |  |  |  | **0.0005** |
| No | 52 ( 75.36 % ) |  | 34 ( 46.58 % ) |  |  |
| Yes | 17 ( 26.64 % ) |  | 39 ( 53.42 % ) |  |  |
| eGFR (ml/min/1.73 m^2^) | 64, 94.27 ± 25.17 |  | 73, 81.34 ± 26.26 |  | **0.006** |
| LDL (mg/dl) | 60, 125.32 ± 51.86 |  | 71, 130.69 ± 38.86 |  | 0.19 |
| MRS | 0, - |  | 65, 0.92 ± 0.92 |  | - |
| Post-stroke time (years) | 0, - |  | 73, 3.14 ± 3.18 |  | - |
| MMSE score | 17, 27 ± 2 |  | 36, 20.58 ± 7.26 |  | **0.002** |
| WMH | 0, - |  | 39, 9.62 ± 5.32 |  | - |
| DAO (ng/ml) | 71, 217.21 ± 83.6 |  | 73, 290.83 ± 111.29 |  | **<0.0001** |

SD: Standard Deviation.

eGFR: Estimated glomerular filtration rate. LDL: low-density lipoprotein.

HTN: hypertension. DM: diabetes mellitus. MMSE: Mini-Mental State Examination. WMH: white matter hyperintensity. DAO: D-amino acid oxidase.

Mann-Whitney U test in 2 groups for continuous data and Chi-square test for categorical data.

Bold values indicate P < 0.05.

**Supplementary Table 2. Uni-variate regression of plasma D-amino acid oxidase (DAO, ng/ml) with all variables.**

| Variable | n | β | S.E. | t | *P*-value |
| --- | --- | --- | --- | --- | --- |
| Group |  |  |  |  |  |
| PSD/ref. PSNoD | 144 | 94.12 | 24.73 | 3.81 | **0.0002** |
| NC/ref. PSNoD | 144 | -47.83 | 17.11 | -2.80 | **0.006** |
| Age | 144 | 3.10 | 0.84 | 3.67 | **0.0003** |
| Gender (Male/ref.Female) | 144 | 31.99 | 19.13 | 1.67 | 0.097 |
| HTN (+) | 142 | 67.49 | 18.26 | 3.70 | **0.0003** |
| DM (+) | 142 | -7.97 | 18.20 | -0.44 | 0.662 |
| eGFR | 137 | -2.06 | 0.30 | -6.94 | **1.47🞨10^-10^** |
| LDL (mg/dl) | 131 | 0.02 | 0.21 | 0.08 | 0.939 |
| MMSE score | 53 | -5.75 | 2.07 | -2.78 | **0.008** |
| Post-stroke time (years) | 73 | 7.33 | 4.06 | 1.81 | 0.075 |
| WMH | 39 | 6.32 | 3.29 | 1.92 | **0.063** |

β: regression coefficient; S.E.: standard error of regression coefficient;

t: the test statistics on slope of the regression line;

Bold values indicate P < 0.05.

**Supplemental figures and figure legends**


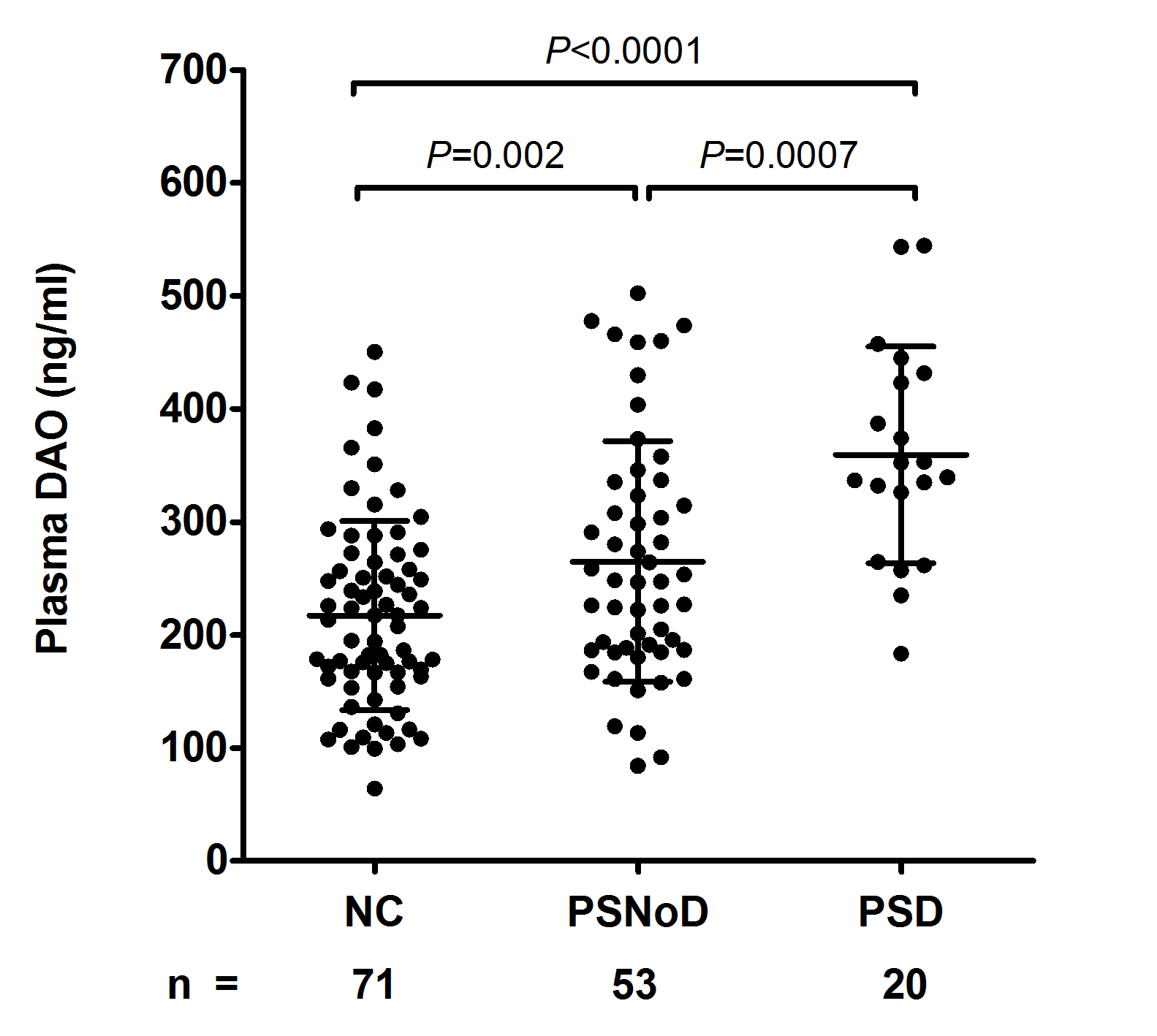


**Supplementary Figure 1.** Plasma D-amino acid oxidase levels (DAO, ng/ml) levels were different between post-stroke without dementia (PSNoD), post-stroke dementia (PSD) patients and normal controls (NC), adjusted for age and gender.
